# Identification of plasmid-mediated tigecycline resistance *tet*(x4) and New Delhi metallo-β-lactamase (NDM) in an *Escherichia coli* isolate from Canada

**DOI:** 10.1128/spectrum.00976-25

**Published:** 2025-10-08

**Authors:** Laura F. Mataseje, Nicole Lerminiaux, Allison McGeer, Jennie Johnstone, Susan Poutanen, Erin McGill, Yves Longtin, on behalf of Canadian Nosocomial Infection Surveillance Program (CNISP)

**Affiliations:** 1 National Microbiology Laboratory 85072 , Winnipeg, Manitoba, Canada; 2 Mt Sinai Hospital, Toronto, Ontario, Canada; 3 Department of Medicine, University of Toronto 7938, Toronto, Ontario, Canada; 4 Public Health Agency of Canada 41687, Ottawa, Ontario, Canada; 5 Jewish General Hospital 5621, Montreal, Québec, Canada; Instituto de Higiene, Montevideo, Canelones, Uruguay

**Keywords:** plasmid, carbapenemase, tigecycline resistance

## Abstract

Rapid spread of carbapenemase-producing Enterobacterales is a global health threat for which tigecycline is one of the few remaining therapeutic options. Resistance by *tet*(x4) is particularly worrisome, given its presence on mobile genetic elements. Here we report on a tigecycline-resistant *Escherichia coli* strain isolated from a patient returning from Pakistan. A *tet*(x4) gene was found on an IncFII plasmid isolated from an ST410 *E. coli* co-harboring NDM-5. The combination of carbapenemase and *tet*(x4) is concerning.

**IMPORTANCE:**

Tigecycline is one of the few last-resort therapeutic options available for treating multidrug-resistant gram-negative bacterial infections, particularly of interest in carbapenemase-producing isolates. Mobile tigecycline resistance is still relatively rare, especially in North America and among clinical isolates. Here we describe the identification of a high-risk clone (*E. coli* ST410) in Canada that contains the tigecycline resistance gene *tet*(x4) on an IncFII plasmid and *bla*_NDM-5_ on a separate IncFIB plasmid. Here, we highlight a rare resistance gene in combination with a carbapenemase, both of which are plasmid mediated, complicating antimicrobial treatment options.

## OBSERVATION

The rapid increase of extensively drug-resistant (XDR) gram-negative bacteria, particularly carbapenem-resistant Enterobacterales, has made treatment challenging. Tigecycline is one of the few last-resort therapeutic options available for treating multidrug-resistant gram-negative bacterial infections (1). However, the newly emerged plasmid-encoded *tet*(x) and efflux *tmex*CD1-*topr*J1 genes in Enterobacterales encode high-level tigecycline resistance (TIG-R) and pose a threat to its clinical efficacy (2, 3). Plasmid-mediated resistance conferred by *tet*(x) genes was first reported in 2019 from *Acinetobacter* and Enterobacterales of both human and animal origin (3). This publication included the first report of *tet*(x4), which was shown to inactivate all tetracyclines, including tigecycline, eravacycline, and omadacycline. It was isolated from *Escherichia coli* of clinical origin co-harboring New Delhi metallo-β-lactamase (NDM) (3). Since then, a few additional reports have identified *tet*(x4) co-harbored with a carbapenemase and/or mobile colistin-resistant genes (mcr-type) (4–8). Here we report the first case of a plasmid-mediated *tet*(x4) co-located in an NDM-producing *Escherichia coli* isolated from a patient in Canada.

In 2021, a 78-year-old male with diabetes was hospitalized in Ontario, Canada. The patient had recently traveled to Pakistan for a medical procedure. Routine admission screening for carbapenemase-producing organisms was performed by direct plating of a pooled nasal/rectal swab onto extended-spectrum β-lactamase isolation media (Oxoid), followed by meropenem disk diffusion screening and a β-CARB test (Bio-Rad). An *E. coli* isolate confirmed by PCR (Xpert CarbaR, Cepheid) to harbor *bla*_NDM_ and *bla*_OXA-48-type_ was recovered and sent to the National Microbiology Laboratory, where antimicrobial susceptibility testing by Sensititre and whole genome sequencing using both the Illumina NextSeq Platform (Illumina, San Diego, USA) and Oxford Nanopore Technologies (ONT) (Oxford, Oxfordshire, UK) Minion platform were performed. Short-read libraries were created with TruSeq Nano DNA HT sample preparation kits (Illumina) and run on an Illumina NextSeq platform. Illumina reads had adaptors trimmed and were filtered for an average Q-score >30 with TrimGalore v.0.6.7 (https://github.com/FelixKrueger/TrimGalore). Long-read sequences were generated using the Rapid Barcoding Kit (SQK-RBK004) on R9.4.1 flow cells. Read data were basecalled and demultiplexed with Guppy v.6.5.7 using the Super High Accuracy model (ONT). Hybrid assemblies were generated with Unicycler v.0.5.0 (https://github.com/rrwick/Unicycler) using default settings. Illumina sequence data and hybrid assemblies were deposited under BioProject PRJNA1240299. StarAMR v.0.10.0 (9) was used to detect antimicrobial resistance genes, plasmid replicons, and sequence type (STs), and MOB-suite v.3.1.4 (https://github.com/phac-nml/mob-suite) was used to assess plasmid mobility. The original culture was mixed with two colony morphologies. Two carbapenemase-producing *E. coli* isolates were recovered: A21005 harboring OXA-48-type and NDM and A21005-2 harboring NDM. Susceptibility data showed near-identical profiles with the exception of TIG-R in A21005-2 (Table 1). Both isolates were non-susceptible to all drugs tested, with the exception of aminoglycosides, plazomicin, fosfomycin, and cefiderocol, making both isolates XDR (10). Complete closed genomes and plasmids were used to examine AMR genes and plasmids between the two *E. coli* (Table 2). The first isolate (A21005) belonged to the high-risk clone ST 361. It contained seven plasmids ranging in size from 6 to 176 kb. Of note, it contained two different *bla*_CTX-M-15_ plasmids, a *bla*_CMY-42_ plasmid, a *bla*_OXA-232_ plasmid, and a *bla*_NDM-5_ plasmid. The second case (A21005-2) belonged to ST410 and harbored four different plasmids, of which a separate *bla*_CMY-146_ plasmid, *bla*_NDM-5_ plasmid, and *tet*(x4) plasmid were observed (Table 2). We looked at plasmid content between isolates to see if any possible plasmid exchange had occurred. Both isolate A21005 and A21005-2 contained some similar plasmid content including a non-AMR 176 kB plasmid [*repA*(pKOX)]; however, the sizes of all other plasmids between the two isolates of the same replicon differed (Table 2). Indeed, the *bla*_NDM-5_ gene was found on an IncF-type plasmid in both isolates, but these plasmids do not appear to be related (<50% identity) and are likely separate acquisitions (data not shown). *E. coli* ST410 is known as a high-risk clone and has been reported worldwide (11). The ST410 lineage has been shown to cause recurrent infections in humans, as well as hospital outbreaks (11).

**TABLE 1:** Antimicrobial susceptibilities (mg/L) for strains tested in this report determined using Sensititre panel CAN1MSTF^*a*^

Drug or test	A21005	Interpretation	A21005-2	Interpretation
Aztreonam	**>16**	**R**	**>16**	**R**
Cefepime	**>16**	**R**	**>16**	**R**
Ceftazidime	**>16**	**R**	**>16**	**R**
Ceftriaxone	**>32**	**R**	**>32**	**R**
Ceftazidime/avibactam	**>16**	**R**	**>16**	**R**
Ciprofloxacin	**>2**	**R**	**>2**	**R**
Colistin	≤1	I	≤1	I
Doxycycline	**>8**	**R**	**>8**	**R**
Ertapenem	**>2**	**R**	**>2**	**R**
Ceftolozane/tazobactam	**>8**	**R**	**>8**	**R**
Gentamicin	≤2	S	≤2	S
Imipenem/relebactam	**>8**	**R**	**>8**	**R**
Levofloxacin	**>4**	**R**	**>4**	**R**
Meropenem	**>16**	**R**	**16**	**R**
Meropenem/vaborbactam	**>8**	**R**	**>8**	**R**
Minocycline	8	I	**>8**	**R**
Piperacillin/tazobactam	**>64**	**R**	**>64**	**R**
Plazomicin sulfate	≤1	S	≤1	S
Tigecycline	≤0.5	S	**4**	**R**
Tobramycin	≤2	S	≤2	S
Trimethoprim/sulfamethoxazole	**>4**	**R**	**>4**	**R**
Fosfomycin agar dilution	1	S	0.5	S
Cefiderocol disk test	17 mm	S	19 mm	S

^*a*^ Bold formatting indicates resistant phenotype according to CLSI (M100:2023). S, susceptible; I, intermediate; R, resistant.

**TABLE 2:** Summary of genotypic data on tetracycline-resistant (A21005-2) and tetracycline-sensitive (A21005) cases^*a*^

Isolate	Plasmid name	AMR genes	Plasmid	Sequence type	Plasmid size
A21005		aadA2, aadA5, *bla*CMY-42, *bla*CTX-M-15, *bla*CTX-M-15, *bla*NDM-5, *bla*OXA-232, *bla*TEM-1B, dfrA12, dfrA17, mph(A), qacE, qacE, qnrS1, sul1, sul1, tet(B)	ColKP3, IncFIB(pB171), IncFII, IncI(gamma), IncY, *repA*(pKOX)	361	n/a^*b*^
	pA21005_A	None	*repA*(pKOX)	n/a	176,160
	pA21005_B	*bla*CTX-M-15, qnrS1	Unknown	n/a	91,863
	pA21005_C	None	IncY	n/a	89,633
	pA21005_D	aadA5, *bla*CTX-M-15, *bla*TEM-1B, dfrA17, mph(A), qacE, sul1, tet(B)	IncFIB(pB171)	n/a	87,008
	pA21005_E	aadA2, *bla*NDM-5, dfrA12, qacE, sul1	IncFII	n/a	82,339
	pA21005_F	*bla*CMY-42	IncI(gamma)	n/a	47,019
	pA21005_G	*bla*OXA-232	ColKP3	n/a	6,141
A21005-2		aadA2, *bla*CMY-146, *bla*NDM-5, *bla*TEM-215, *bla*TEM-215, dfrA12, floR, mph(A), qacE, qepA4, sul1, tet(X4)	IncFIB(pB171), IncFII, IncI(gamma), *repA*(pKOX)	410	n/a
	pA21005-2_A	None	*repA*(pKOX)	n/a	176,160
	pA21005-2_B	aadA2, *bla*NDM-5, *bla*TEM-215, dfrA12, mph(A), qacE, qepA4, sul1	IncFIB(pB171)	n/a	93,954
	pA21005-2_C	*bla*TEM-215, floR, tet(X4)	IncFII	n/a	87,663
	pA21005-2_D	*bla*CMY-146	IncI(gamma)	n/a	24,054

^*a*^ Total content is shown for each isolate followed by each plasmid.

^*b*^ n/a, not applicable.

**Fig 1:**
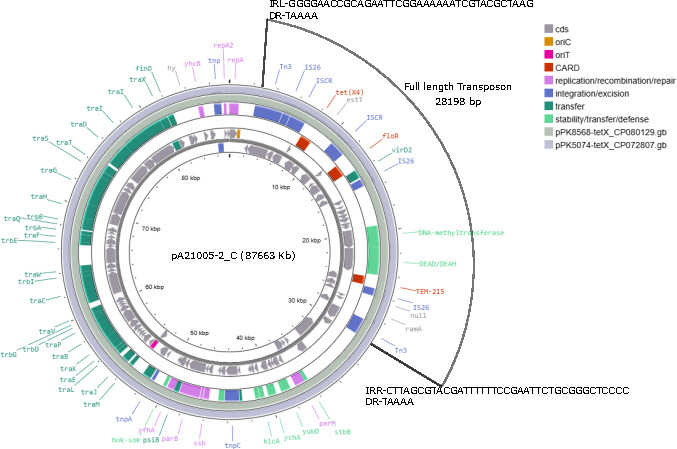
Plasmid generated with Proksee. Innermost two tracks represent forward and reverse open reading frames of pA21005-2. The middle two tracks are annotations generated by CARD RGI and mobileOG databases. The outer two tracks show comparisons to other *tet*(x) plasmids isolated from chickens in Pakistan using BLAST.

The *tet*(x4) gene was harbored on a 87 kb IncFII plasmid (p0821005-2_C) that also contained the resistance genes *flo*R and *bla*_TEM-125_. (Fig. 1). This plasmid was predicted to be conjugative by MOB-suite with a MOBF relaxase and MPF_F mating pair formation protein. This plasmid encoded the *hok-sok* Type I toxin-antitoxin system, antirestriction protein KlcA, single-stranded binding protein Ssb, an SOS inhibitor protein PsiB, and several genes involved in plasmid stability (*stbB* and *parM*). Similar plasmids have been observed from *E. coli* isolated from chickens in Pakistan (8) as shown in the figure. Other studies have reported the *tet*(x4) gene on various other plasmid backbones including IncX (4, 12), IncQ (5, 6), and hybrid plasmids (4, 12). Though some studies have characterized the genetic environment by types (I and II) (12) or by groups (G1–G4) (4), the complete mobile elements that carry *tet*(x4) have not been fully described. Here we observed Tn*3*-IS*26*-IS*CR-tet*(x4)-est*T*-IS*CR-floR-virD2*-IS*26-bla*TEM-215-IS*26-ramA-*Tn*3* among several hypothetic open reading frames, as well as a DNA-methyltransferase and a restriction endonuclease (DEAD/DEAH). The duplicated Tn*3* was flanked by 38 bp inverted repeats and a 5 bp target site duplication, suggesting this is a 28,198 bp mobilizable fragment (Fig. 1). Though the *E. coli* ST361 also harbored an IncFII plasmid, these were only 70% similar to each other and did not share similar AMR genes.

In summary, we observed a high-risk *E. coli* clone that harbored multiple AMR plasmids, including genes for tigecycline and carbapenemase resistance, overall, making it XDR. To the authors’ knowledge, this is the first report of a plasmid-mediated tigecycline resistance gene detected in Canada. Its occurrence on a plasmid with other resistance genes, including a carbapenemase, is of significant concern.
